# Beyond the Granuloma: New Insights into Cardiac Sarcoidosis Using Spatial Proteomics

**DOI:** 10.21203/rs.3.rs-4289663/v1

**Published:** 2024-05-07

**Authors:** Eliot Peyster, David Smith, Therese Bittermann, Paco Bravo, Kenneth Margulies

**Affiliations:** University of Pennsylvania; Children’s Hospital of Philadelphia; University of Pennsylvania; University of Pennsylvania; University of Pennsylvania

## Abstract

Cardiac sarcoidosis is poorly understood, challenging to diagnose, and portends a poor prognosis. A lack of animal models necessitates the use of residual human samples to study sarcoidosis, which in turn necessitates the use of analytical tools compatible with archival, fixed tissue. We employed high-plex spatial protein analysis within a large cohort of archival human cardiac sarcoidosis and control tissue samples, studying the immunologic, fibrotic, and metabolic landscape of sarcoidosis at different stages of disease, in different cardiac tissue compartments, and in tissue regions with and without overt inflammation. Utilizing a small set of differentially expressed protein biomarkers, we also report the development of a predictive model capable of accurately discriminating between control cardiac tissue and sarcoidosis tissue, even when no histologic evidence of sarcoidosis is present. This finding has major translational implications, with the potential to markedly improve the diagnostic yield of clinical biopsies obtained from suspected sarcoidosis patients.

## Introduction

Sarcoidosis is a multi-system inflammatory disease of uncertain cause and with widely ranging clinical manifestations. Cardiac involvement is considered the disease’s most serious manifestation, conferring a high risk of heart failure, life-threatening arrhythmias, and all-cause mortality.^1–3^ The exact prevalence of cardiac sarcoidosis (CS) is difficult to assess due to a significant burden of occult disease and missed diagnoses,^2,4,5^ with many cases only diagnosed after sudden cardiac death^6^ or at the time of heart transplantation/left ventricular assist device (LVAD) implantation.^7,8^

Though incurable, CS is a treatable disease. The mainstay of therapy is systemic immunosuppression, initially using high-dose glucocorticoids, followed by a transition to systemic steroid-sparing agents.^2,3^ However, because of the potential risks of systemic immunosuppression, current guidelines only recommend initiating treatment in patients with definitive, active CS. Owing to the particularly high risk of life-threatening arrhythmias in the CS population,^9^ guidelines for primary prevention implantable cardiac defibrillator (ICD) make special mention of CS, recommending ICDs for many CS patients even when left ventricular ejection fraction (LVEF) is relatively preserved. However, because ICD therapy has known risks,^10^ these recommendations are only applicable to patients with definitive CS diagnoses.

Given the high morbidity of CS, making a timely and accurate diagnosis is of paramount importance. In general terms, diagnosis relies on clinical characteristics, tissue sampling, and exclusion of alternative causes. More specifically, the diagnostic reference standard for CS is histologic identification of non-caseating granulomas on endomyocardial biopsy (EMB) tissue.^1,3,4^ Unfortunately, due to the heterogeneous and sparse distribution of these pathognomonic granulomas, the true diagnostic yield of EMB is estimated at 25%.^2,11,12^ Advanced imaging techniques such as cardiac MRI and FDG-PET have value in CS as both screening tests and treatment-surveillance tests, but remain most useful as ancillary diagnostics due to limitations of their sensitivity and specificity.^1^

The high incidence of occult disease, risky and un-targeted treatment regimens, and insensitive diagnostic gold standard each highlight key areas of unmet need in CS. Without a known cause for sarcoidosis, development of animal models to study this disease has not been possible. Thus, researchers are forced to rely on human biosamples as the primary source for diagnostic, mechanistic, and therapeutic investigation. Until recently, tools for examining these residual or archival human tissue samples have been limited, which in turn has limited breakthroughs in translational CS research. In this manuscript, we describe the application of high-plex spatial protein analysis within a large cohort of archival human CS tissue samples. The present study differs from prior CS research^13^ by leveraging the full potential of spatial-omics technology to individually study the myocytes, stromal cells, and vascular cells *beyond* the pathognomonic granulomas that define the disease. This approach is motivated by the fact that although granulomas are the hallmark of CS, they occupy only a small fraction of the total cardiac tissue area. We therefore hypothesize that key patient outcomes like cardiac pump failure and arrhythmia burden are a result of tissue-level biology which occurs far-removed from the regions of overt, active CS inflammation. Using this novel conceptual framework and cutting-edge spatial technology, we describe novel CS biology and uncover a concise set of tissue-level biomarkers which provide high diagnostic accuracy even in tissue without pathognomonic granulomas.

## Methods

### Cohort Description:

The study cohort consisted of n = 48 formalin-fixed, paraffin embedded (FFPE) cardiac tissue samples from the University of Pennsylvania. N = 39 tissue samples were derived from patients with tissue confirmed CS, n = 14 of which were EMBs obtained during diagnostic workup early in CS disease course, and n = 25 were ‘advanced-stage’ CS tissue obtained at time of cardiac transplant or LVAD implant. Although all CS patients contributing samples had tissue-confirmed CS, among the specific tissue blocks used for this study, n = 24 had pathologist-documented granulomatous inflammation on the tissue sample used in the study cohort, n = 7 had documented inflammation of uncertain etiology (ranging from “occasional, minimal inflammatory infiltrates” to “significant lymphocytic inflammation”), and n = 8 had no identified inflammation or granulomas. The remaining n = 9 study tissue samples represented Control cases, consisting of n = 3 ‘heart failure controls’ with known, non-inflammatory, non-ischemic cardiomyopathy (NICM), n = 4 ‘non-failing (NF) controls’ sourced from declined heart donors who had suffered cardiac arrests, and n = 2 ‘inflammatory controls’ sourced from heart transplant recipients with no significant acute rejection but with chronic allograft dysfunction (TXP). A diverse, heterogeneous, cohort of ‘non-sarcoid’ controls was selected specifically to facilitate an understanding of which protein expression markers are particularly altered in CS, as these would represent the optimal candidate biomarkers for future efforts to improve CS diagnostic accuracy. Cohort details are presented in Table 2. Procurement of human myocardial tissue was performed under protocols and ethical regulations approved by Institutional Review Boards at the University of Pennsylvania and the Gift-of-Life Donor Program (Philadelphia, Pennsylvania, United States), and complies with the Declaration of Helsinki.

### Sample Processing and Immunofluorescence Staining:

Unstained sections from FFPE samples were cut (6 μm thickness) and mounted on glass slides. Four sections were mounted on each slide to enable more efficient utilization of study reagents and machine time. In order to permit digital pathology examination and segmentation, study slides underwent immunofluorescence staining for the vascular endothelium marker CD31 (Abcam ab215912, Cambridge UK), the cardiomyocyte marker Troponin I (Abcam ab196384, Cambridge UK), and the stroma/collagen marker Collagen VI (Abcam ab207292, Cambridge UK), along with SYTO for pan-nucleic acid staining (Thermo Fisher S11363, Waltham MA) prior to loading into the GeoMx instrument. Whole-slide scanning was performed by the NanoString GeoMx Digital Spatial Profiler (DSP) instrument.

### Digital-Spatial Protein Expression Analysis:

#### GeoMx DSP Instrument

Spatial protein expression analysis was conducted using the Nanostring GeoMx DSP instrument (Nanostring Technologies, Seattle, WA). Briefly, this instrument relies on photo-cleavable oligonucleotide barcodes which are conjugated to antibodies for the desired protein targets. After incubating the study immunofluorescence slides in the study antibody/oligonucleotide panel reagents, the slides are digitized using the GeoMx DSP instrument to produce 40x magnification (0.25microns-per-pixel resolution) digital images. Regions of interest (ROIs) from within the digitized slides are then selected for analysis, followed by application of focused UV light to cleave oligonucleotide barcodes. The digital micro-mirror device of the DSP instrument tunes the UV light with 1-micron resolution, allowing for great flexibility and specificity in ROI selection. Released tags are collected by micro-capillary aspiration and stored on nCounter optical barcodes (Nanostring Technologies) for sequencing-based quantitation which is registered to the specific ROIs of data collection.

Methods for sample preparation prior to running DSP analysis and for cartridge and sequencing after DSP analysis are described in the supplemental methods.

### Protein Expression Panel:

The study protein panel for the GeoMx spatial protein expression analysis consisted of n = 79 protein targets designed to provide detailed descriptions of CS stromal cell phenotypes, cell proliferation status, immune cell population, immune cell activation status, immune checkpoint activity, pro- and anti-apoptotic factors, mitogen-activated protein kinase (MAPK) pathway activity, and phosphoinositide-3-kinase–protein kinase (PI3K/AKT) pathway activity (see Supplemental Table S.1 for detailed list).

### ROI Selection and Spatial Analysis Workflow:

Tissue ROIs were selected to assess protein expression changes with increasing distance from the CS-defining granulomas. To that end, ROIs were selected from within granulomas, from peri-granulomatous cardiac parenchymal tissue (< 300μm from granulomas), and from granuloma-remote parenchymal tissue (> 500μm away from the edge of any detected granuloma region) in each tissue sample whenever possible. Granuloma-remote ROIs were all ~ 600×600μm squares, and based on the 500μm distance requirement between ROI edge and the nearest granuloma, each granuloma-remote ROI represents the center of a granuloma-free tissue footprint that is ~ 1.3×1.3cm. This granuloma-free footprint is comparable to the size of a standard clinical EMB, and thus simulates a ‘false negative’ biopsy using conventional histologic methods for diagnosing CS.

**Figure 1:**
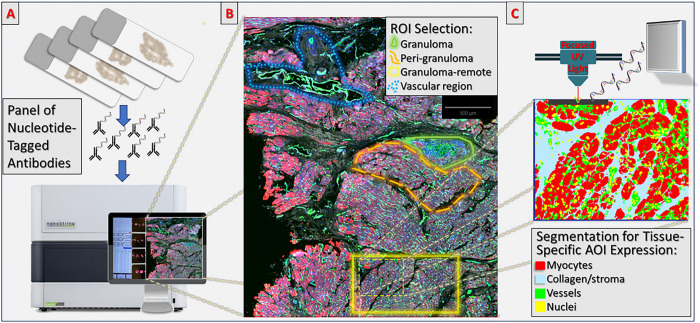
GeoMx workflow. Panel A: after 4-plex immunofluorescence staining with CD31 for vessels, Troponin I for myocytes, Collagen VI for collagenous stroma, and a pan-nuclei stain, study slides are bathed in nucleotide-tagged antibodies for each of the protein panel markers and loaded into the GeoMx DSP instrument. Panel B: Region of interest (ROI) selection as per study experimental design, focusing on granuloma ROIs and a series of cardiac parenchyma ROIs corresponding to ‘peri-granuloma-regions, ‘granuloma-remote’ regions, and large vascular bed regions. Panel C: Prior to expression data collection, digital pathology image segmentation of parenchyma ROIs is performed to divide ROIs into tissue-compartment-specific ‘Areas of Illumination’ (AOIs). AOIs representing the Myocyte compartment, the Stroma compartment, and the Vascular compartment are digitally segmented using pixel intensity thresholds, hole-filling, and dilation operations. Finally, focused ultraviolet light from the GeoMx instrument is used to cleave nucleotide barcodes and sequentially extract protein expression data from each AOI in each ROI.

The spatial analysis workflow leveraged established methods for digital pathology image analysis,^14–17^ focusing not only on selecting ROIs at different distances from granulomas, but also on conducting a ‘tissue-compartment-specific’ analysis within each individual cardiac parenchymal ROI. Specifically, our approach permitted discrete segmentation of parenchymal ROIs (e.g. non-granuloma ROIs) into distinct *areas of illumination* (AOIs), with AOIs labeled according to the segmented tissue-types contained within them: cardiomyocyte AOIs, interstitial stroma AOIs (including fibroblasts, immune cells, capillaries/small vessels etc.), and the vascular compartment AOIs (containing larger vessels along with perivascular stroma). Segmentation was conducted using native GeoMx software by customizing pixel-intensity thresholds in each ROI for the different morphologic immunofluorescence antibodies used (CD-31 for vessels, Troponin-I for myocytes, Collagen-VI for collagenous stroma, SYTO for nuclei). By achieving accurate segmentation of each tissue-compartment, we were able to measure protein expression separately for each tissue compartment AOI within an ROI, allowing more nuanced assessments of tissue content. Figure 1 provides a summary of the study workflow with visual examples of ROIs and AOIs.

It is important to note that image segmentation designations reflect the predominant, but not exclusive, constituents of each compartment. Myocyte AOIs typically contain not only myocytes, but also small, adjacent areas of stromal tissue which do not stain significantly for collagen VI (and therefore were not easily segmented into the collagenous stroma class). Stromal AOIs typically include small venules and capillaries. As a result, the reported protein expression data for myocyte AOIs will include some non-myocyte proteins while stromal AOIs will also contain endothelial cell proteins. Because myocytes dominate in terms of cell count and area within a myocyte AOI, protein expression data for proteins expressed in *both* myocytes and stromal cell populations will be largely attributable to the myocyte cell contribution. However, for proteins *not* typically expressed by myocytes, expression within myocyte AOIs will in fact be attributable to stromal cell types. From a data presentation perspective, myocyte AOI results in this manuscript will *only* include proteins with known expression in myocytes, based on review of published and unpublished single cell RNA^18,19^ and proteomics datasets^20,21^ (refer to Table 1). However, no data will be discarded through this process, since every study analysis also includes results at the full-ROI level which incorporates *all* protein expression data from within the ROI.

### Experimental Design:

Our methodologic goal was to leverage the native functions of the GeoMx platform and established digital pathology image analysis methodology to perform a comprehensive and highly nuanced assessment of cohort tissues. We sought to explore several facets of CS biology by executing comparisons within and between tissues in the CS tissue cohort, and executing comparisons between CS tissues and the non-CS control cohort. Specific pre-specified analyses for the cohort of CS patients alone included: **1)** analysis of differentially expressed proteins (DEPs) and protein expression variation between granuloma ROIs of CS patients, **2)** analysis of DEPs between biopsy and explant samples from CS patients to identify distinctions between earlier vs. later disease, **3)** analysis of DEPs between ‘peri-granuloma’ ROIs and ‘granuloma-remote’ ROIs to assess whether there is a ‘distance gradient’ in protein expression, and **4)** analysis of DEPs between granuloma-free ROIs which contain inflammatory infiltrates and those without infiltrates. Pre-specified analyses for comparing CS tissues to non-CS Controls focused solely on an analysis of granuloma-remote, inflammatory-infiltrate-free ROIs, comparing these histologically bland CS ROIs to the diverse control cohort ROIs. The goal of this analysis was to uncover protein expression patterns that may be specific to CS, and which are more homogenously distributed than classical granulomatous inflammation. Using the protein expression data from this comparison of CS to Controls, exploratory predictive modeling was performed to assess the diagnostic potential of using in-situ protein biomarkers.

#### Data analysis

All analyses were conducted with R 4.2.3, Stata IC 15.0, and Python 3.10.13.

#### Data Visualization

Unbiased data visualizations were generated for each analysis described above. Principal component analysis (PCA) and t-distributed Stochastic Neighbor Embedding (t-SNE) were utilized for data visualization.

### Differential Expression Analysis:

Probe counts were processed, and their quality assessed using GeoMxWorkflows (v1.8.0), NanoStringNCTools (v1.10.0), and GeoMxTools (v3.5.0). Briefly, segments (AOIs) were filtered based on nuclei count, binding density, surface area, and background signal. Among all categories, 5 of 571 segments were flagged for low nuclei count and discarded. All probes (79) were retained. Inspired by the work of van Hijfte et al.^22^, several normalization strategies were considered and evaluated based on the correlation of mean expression and p-value as calculated from sarcoid vs. control. The lowest correlation and best symmetry detected with significant probes (i.e. least bias towards either condition) was observed with quantile normalization. For the ROI-level and AOI-level expression data, differentially expressed probes were identified using mixed linear model. Starting from a full parameterization of

$$\log\left(quantile\;\left(\left.\left.{probe}_{i}\right)\right)\right.\right.={sarcoid}_{i}+{infiltrate}_{i}+{explant}_{i}+{distance}_{i}+(1|donorID)$$

Where sarcoid is defined whether the donor was clinically diagnosed with sarcoidosis, infiltrate is the presence of infiltrate, explant is whether the tissue was collected as biopsy or from explant tissue, and distance is an ordinal measure of distance from the edge of the nearest granuloma. Every permutation of each reduced model was fit to find the optimal model based on ANOVA against the full model, and we found the full model to perform best for most probes.

For analysis of myocytes, we noticed improbably high signal of common immune cell markers. We suspected this signal arose from inefficient segmentation resulting in stromal contamination. We used a method like that implemented by AUCell2 to score the signal of immune markers (CD68, CTLA4, CD3, CD4, PD-1, CD8, CD45, and CD20).^23^ Myocyte segments with a score > 0.2 were dropped from analysis of myocyte AOIs (10/234); meaning, > 20% of the immune markers were found in the top 20%ile of expressed probes. Despite dropping these myocyte segments, data from these AOIs was retained for full-ROI-level analyses.

### Ordinal Logistic Regression:

To further assess effects of distance from CS inflammation on parenchyma tissue ROI/AOI expression, ordinal logistic regression was performed for all study panel markers. The ordinal scale was designed to capture relative distance, with Inflammation(−) peri-granuloma ROIs were assigned a value of ‘1’, inflammation(−) granuloma-remote ROIs assigned a value of ‘2’, and Control ROIs assigned a value of ‘3’.

#### Sarcoid classifier models

To predict a diagnosis of sarcoid vs. non-sarcoid, we used either the full-ROI-level data or the individual myocyte and stroma AOI-level data. The primary objective of the modeling experiment was to generate a parsimonious model for predicting CS, because the long-term translational goal is to use this model to inform the development of a multiplex immunofluorescence (IF) platform (which are typically limited to ~ 7 markers).^16^ Because of specific interest in utilizing quantitative multiplex IF in future applications, the feature set was specifically curated prior to statistical modeling in order to exclude markers which are not well suited to quantitative IF. For example, ubiquitously expressed proteins involved in canonical pathways are generally poorly suited to this type of work, while cell-surface markers and cell type-specific enzymes and transcription factors are particularly well suited. An additional initial requirement for consideration in the modeling experiment was whether a variable was consistently increased or decreased across the three control subgroups as compared to CS. Due to deliberate and marked heterogeneity of our control population, and due to a desire to identify the proteins with the most convincing and specific differential expression in CS, this criterion was felt reasonable as a means of ensuring good model fit and generalizability. In total, n = 54 variables were considered at the start of modeling work. The potential variables considered during statistical modeling are outlined in Supplemental Table S.2.

The specific modeling task was to predict whether a granuloma-remote, inflammation(−) ROI belonged to a CS case vs. a Control case. The study cohort of ROIs meeting these criteria was divided into a training set (67% of the ROIs, n = 103) and a held-out testing set (33% of ROIs, n = 51). Feature selection and model training was conducted on the training set, with the final derived model performance assessed on the held-out testing set. Three approaches were assessed for the classification task: 4-fold Cross-validated LASSO (LassoPack, Stata IC 15.0 & PyTorch v2.1.1), support vector classifier (PyTorch v2.1.1), and gradient boosted trees (scikit-learn v1.3.2). For models trained on the segmented myocyte and stroma data, the first learnable step was a linear regression with the two segments. For the LASSO model, we found optimal performance with α = 0.001. The support vessel classifier model used a RBF kernel with a log softmax output layer. The gradient boosted model was fit using 50 trees with a max depth of 3. Performance was overall similar between the three methods prior to further feature reduction to meet the pre-specified limit of six or viewer variables. Therefore, LASSO was ultimately utilized due to simplicity and broad acceptance. Starting with LASSO-selected features, the total feature set was then reduced variables via backwards, stepwise, leave-one-out cross validated logistic regression until optimal performance at seven or fewer variables was achieved. For the logistic regression, optimal model classification cut-point was determined via the Liu method.^24^ Model performance was assessed in the held-out test set via accuracy, area under the receiver-operator curve (AUC), sensitivity, specificity, positive predictive value and negative predictive value.

## Results

### Cohort summary:

The study cohort is summarized in Table 1. Briefly, the baseline characteristics of the CS cohort differed from the Control cohort in the proportion of female patients (13% for CS group vs. 50% for controls, p = 0.012), and in the average LVEF at tissue sampling (26.5% for CS group vs. 39.7% for controls, p = 0.03). There were no significant differences among baseline characteristics of CS patients contributing earlier-disease-stage biopsy samples to the cohort vs. those contributing advanced-stage disease samples.

The GeoMx workflow was deployed across the entire 48 sample cohort, generating 305 ROIs which yielded a total of 521 AOIs after segmentation of tissue compartments in cardiac parenchyma ROIs. For final analyses, there were n = 39 granuloma ROIs, n = 227 non-granulomatous cardiac parenchyma ROIs (from which myocyte and stroma AOIs were segmented), and n = 39 large vascular bed ROIs (from which vascular AOIs were derived – see Fig. 1). Among parenchymal tissue (non-granuloma) ROIs in the CS cohort, there were n = 62 ROIs from EMB tissues (n = 8 peri-granuloma parenchymal ROIs, n = 54 granuloma-remote parenchymal ROIs), and n = 161 from advanced-stage explant tissues (n = 19 peri-granuloma parenchymal ROIs, and n = 113 granuloma-remote parenchymal ROIs, and n = 29 large vascular bed ROIs). There were n = 35 ‘inflammation(+)’ parenchymal ROIs in the CS cohort which, despite not containing granulomas, had overt histologic immune cell infiltration. There were n = 43 ROIs from Control tissues, including n = 33 parenchymal ROIs and n = 10 large vascular bed ROIs.

### Spatial protein expression results demonstrate substantial CS granuloma heterogeneity, both within and between patients:

For most study panel proteins (49/79, 62%), the variance in protein expression between granuloma-containing ROIs was higher than the variance between non-granuloma parenchymal ROIs. This is notable, considering parenchymal ROIs consist of stroma and myocytes from hearts with widely varying LVEFs and which, in some cases, contain overt interstitial inflammatory cell infiltrates. The 10 most variably expressed proteins among granuloma ROIs are predominantly immune cell-type markers for macrophages, T-cells, granulocytes and antigen presenting cells: CD68, HLA-DR, CD11c, CD45, CD3, IDO1, CD44, CD40, CD66b, and BCL6.

There were only modest differences in the granuloma protein expression between ‘advanced-stage disease’ tissues and EMB tissues obtained earlier in the disease course. Immune checkpoint molecule Tim-3 and activated fibroblast marker FAP-alpha have significantly increased expression in advanced-stage CS hearts (p = 0.02 and p = 0.017, respectively). In contrast, immune checkpoint molecule VISTA, nuclear/proliferation marker Histone-H3, and activated MEK1 (part of the RAF/MEK/ERK pathway known to be involved in inflammation and linked to granuloma formation when inhibited)^25,26^ each had significantly increased expression in earlier/active stage CS (p = 0.007, p = 0.013, and p = 0.017 respectively).

We performed a sub-analysis of the nine CS tissue samples which contributed *multiple* granuloma ROIs to the dataset. The PCA biplot in Supplemental Figure S.1 suggests substantial *intra*-sample heterogeneity in CS granulomas, with only modest within-sample groupings. Nearest-neighbor analysis of the PCA plot demonstrates that for cases contributing multiple granuloma ROIs, the closest-clustering granuloma ROI is more likely to be from a *different* tissue sample than from the same sample (13/23, 56.5%). At the individual protein level, the *intra*-sample coefficient of variation (COV) exceeded *inter*-sample COV for an average of 11 protein markers in each multi-granuloma sample, with the most substantial intra-sample variation seen in T-cell and cytotoxic cell markers (CD27, CD8, GZMB), apoptotic markers (CD95/FAS, Cleaved-Caspase-9), and checkpoint molecules (PDL2) (supplemental Table S.3).

### Analysis of the Cardiac Parenchyma Highlights the Protein Expression Profiles of Active and ‘Burnt Out’ CS:

**Figure 2:**
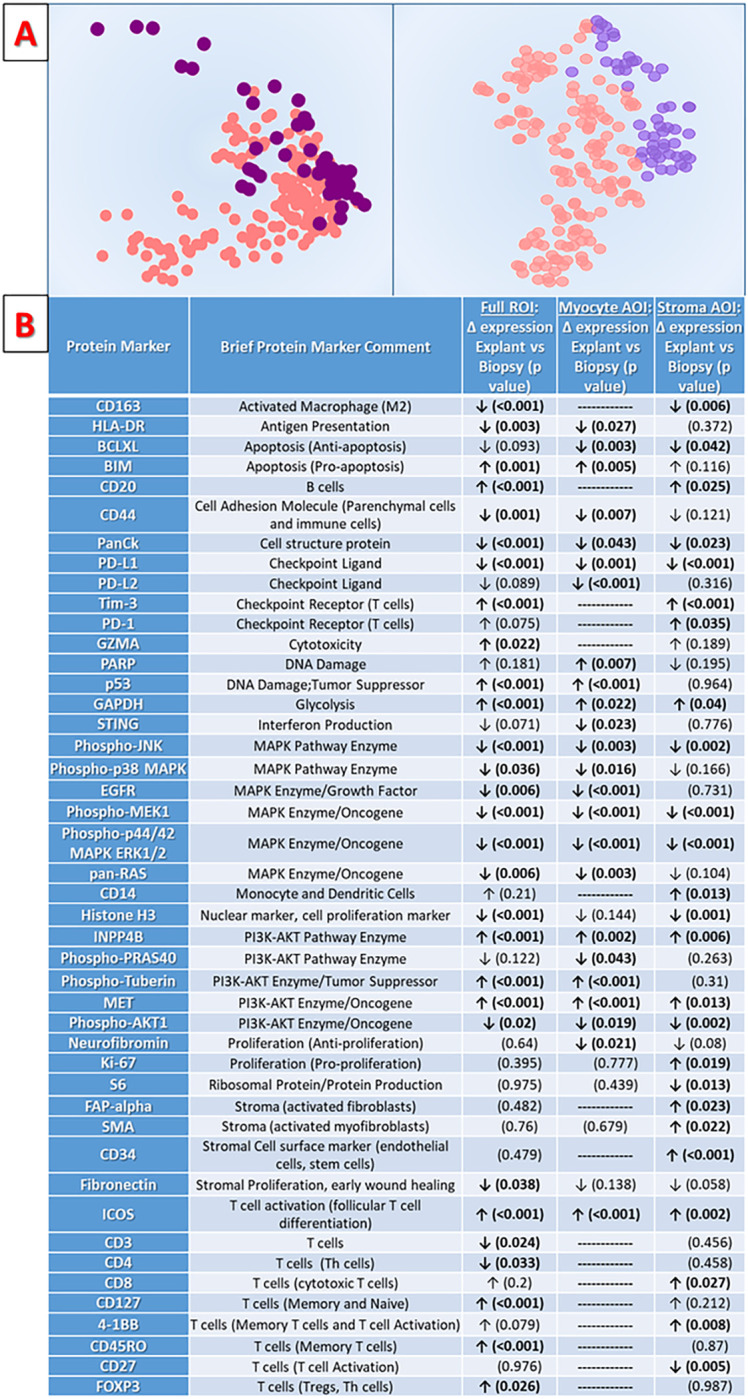
Cardiac sarcoidosis (CS) disease stage analyses, comparing spatial protein expression between biopsy tissue samples obtained early during disease management and advanced-stage disease samples obtained at cardiac explant. **2a**: Principal component analysis (PCA, on left) and t-SNE (on right) plots of panel-wide protein expression in the study regions of interest (ROIs). ROIs from biopsy samples obtained earlier in disease (purple circles in PCA and in t-SNE plots) predominantly group together at one edge of the dataset, while ROIs from advanced-stage samples largely occupy the rest of the field (pink circles in PCA and t-SNE plots). **2b**: Tabular results for differential protein expression analyses comparing samples by CS disease stage. ROI-level protein expression for biopsy samples obtained earlier in disease are compared to explant samples obtained in advanced-stage disease. In addition to ‘full-ROI-level’ differential expression results, digital segmentation of study ROIs enables tissue-compartment-specific analysis of protein expression in discrete ‘areas of illumination’ (AOIs). Via this method, cardiomyocyte-specific and stromal-tissue-specific differential protein expression comparisons between biopsy and explant samples are also presented. Differentially expressed proteins with p-values <0.05 are shown for full-ROI level data and for myocyte-specific and stroma-specific data.

Analysis of intrinsic cardiac parenchymal ROIs (e.g. non-granuloma ROIs) demonstrates substantial differences in the expression of immunologic, cell survival, and cell death pathways between EMB tissue samples obtained during the active workup/management phase of CS and advanced-stage disease tissue samples obtained at cardiectomy. These differences are readily apparent via unsupervised data visualization with PCA and t-SNE plots in Fig. 2a. When analyzed as non-compartmentalized ROIs, n = 33 proteins show significant differential expression based on disease stage (Fig. 2b and Supplemental Table S.4). When segmented as compartment-specific AOIs, there are n = 24 significant DEPs in the myocyte compartment, and n = 27 DEPs in the stroma compartment.

While substantial overlap exists between DEPs in the ROI- and AOI-level analyses, AOI analyses add important context. We observed significantly increased pro-apoptotic factors and decreased MAP-kinase and PI3K/AKT pathway activity in the myocyte compartment AOIs of advanced-stage hearts – a finding consistent with prior research on end-stage cardiomyopathy more generally^.27,28^ In stroma compartment AOIs of advanced-stage hearts, we observed significant increases in markers of activated/differentiated fibroblasts (FAP-alpha and SMA). Again, this is consistent with known advanced-stage cardiomyopathy biology.^29^ However, we also observed numerous significant shifts in expression of immune-related protein markers in the stroma and myocyte AOIs when comparing tissues acquired earlier vs. later in disease which are not as easily explained.

in advanced-stage CS, there was a significant decrease in several macrophage and effector T-cell lineage markers (CD3, CD4, CD163), which coincided with significant increases in markers of longer-lasting regulatory T-cell (Treg), memory T-cell and B-cell populations (ICOS, FOXP3, CD45RO, CD127, CD20). In addition, the stroma and myocytes of advanced-stage cases manifest a less ‘immune primed’ state, with decreased expression of major histocompatibility (MHC) molecule HLA-DR, checkpoint molecules PD-L1/PD-L2, and interferon-producing STING. Overall, these findings are consistent with the theoretical biology of the ‘late fibrous phase’ stage of CS (sometimes called ‘burnt out’ CS),^30^ which is thought to involve increased fibrosis along with a decrease in active inflammatory elements.^30^ Our findings support long-standing theories about this process, highlighting numerous key immune cell types and effectors which change as CS progresses.

### Examining the ‘Distance-Gradient’ of Cardiac Parenchymal Biology in CS:

**Figure 3:**
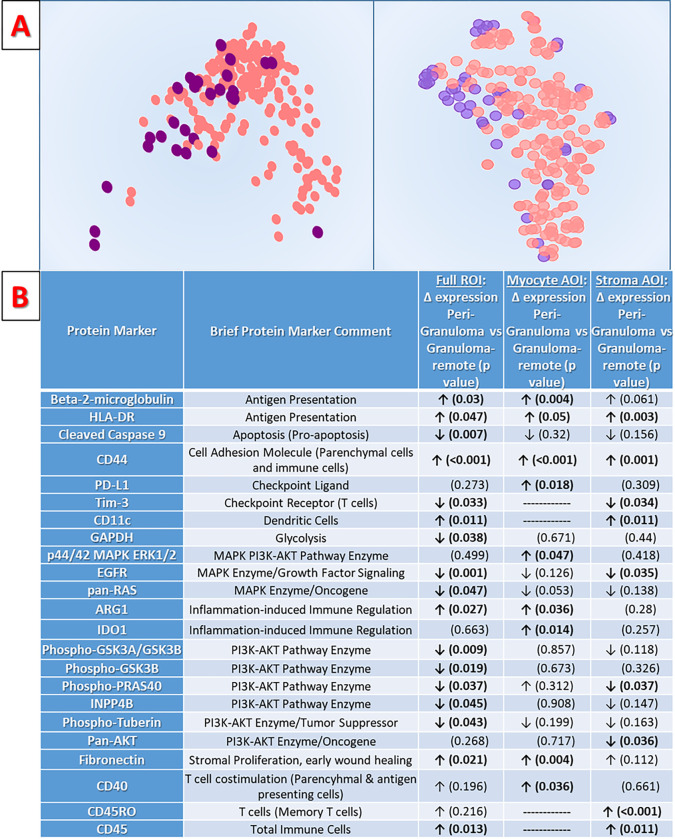
Cardiac sarcoidosis (CS) ‘distance gradient’ analyses, highlighting the impact of proximity to granulomatous inflammation on cardiac parenchyma protein expression. **3a:** Principal component analysis (PCA, on left) and t-SNE (on right) plots of panel-wide protein expression in the study regions of interest (ROIs). ‘Peri-granuloma’ ROIs located within 300μm of a granuloma (purple circles in PCA and in t-SNE plots) predominantly group together on the left edge of the dataset, while ‘granuloma-remote’ ROIs located >500 μm from any granuloma largely occupy the rest of the field (pink circles in PCA and t-SNE plots). **3b:** Tabular results for differential protein expression analyses comparing samples by relative proximity to granulomatous inflammation. ROI-level protein expression from peri-granuloma cardiac parenchyma is compared to ROI-level expression from granuloma-remote cardiac parenchyma. In addition to ‘full-ROI-level’ differential expression results, digital segmentation of study ROIs enables tissue-compartment-specific analysis of protein expression in discrete ‘areas of illumination’ (AOIs). Via this method, cardiomyocyte-specific and stromal-tissue-specific differential protein expression comparisons between peri-granuloma and granuloma-remote parenchymal regions are also presented. Differentially expressed proteins with p-values <0.05 are shown for full-ROI level data and for myocyte-specific and stroma-specific data.

As shown in Fig. 3 and Supplemental Table S.5, spatial analysis of the CS parenchyma reveals a previously unreported ‘distance-gradient’ in protein expression, in which numerous panel proteins were differentially expressed based on a tissue region’s relative distance from granulomas. This distance gradient is apparent with unbiased data visualization via PCA and t-SNE (Fig. 3a), and persists even when accounting for confounders like histologic inflammatory infiltrates during differential expression testing with mixed effects models (Fig. 3b).

We also examined ‘inflammation(+)’ ROIs which *do* have discrete, non-granulomatous, interstitial inflammatory cell infiltrates, comparing these extreme examples of ‘proximity to inflammation’ to ‘inflammation(−)’ ROIs without any discrete inflammation. Unsurprisingly, there were many significant DEPs between these groups, including increased expression of numerous immune effector cell markers: CD3, CD4, CD8, CD68, CD163, GZMA, CD14, and CD45 (Supplemental Table S.6). Interestingly, at the ROI-level, 70% (12/17) of the significant DEPs which were observed during our inflammation-adjusted comparison of peri-granuloma ROIs to granuloma-remote ROIs are also significant DEPs when comparing overt ‘inflammation(+)’ ROIs to ‘inflammation(−)’ ROIs. However, these overlapping DEPs are largely *not* classic immune-effector cell markers, and instead suggest that the distance-gradient observed in this study arises from subtler findings of immune activity.

Expression of most specific immune cell-types do not differ between peri-granuloma and granuloma-remote regions. However, peri-granuloma stroma does have a larger population of total immune cells (CD45+). This is primarily due to significant increases in long-lasting immune ‘sentinels’ in the form of CD11c + dendritic cells and CD45RO + memory T-cells, rather than to increases in classic effector cell-types such as those found in overt ‘inflammation(+)’ parenchyma. Beyond specific immune cell-type markers, peri-granuloma stroma exhibits increased expression of class I and II MHC molecules (Beta-2-microglobulin and HLAD-DR) – a phenomenon known to occur in association with myocardial inflammation.^31–35^ Peri-granuloma stroma also exhibits increased expression of inflammation-associated pro-fibrotic mediators such as arginase 1,^36,37^, fibronectin,^38^ and CD44^39,40^. Interestingly, peri-granuloma stromal cells have decreased expression of immune checkpoint molecule Tim-3, suggesting that that peri-granuloma lymphocytes may be less responsive to immune-checkpoint-mediated inhibition.^41^ Finally, distance-dependent protein expression also impacts cardiomyocyte biology. Peri-granuloma myocytes manifest a ‘stress-activated’ state,^42^ with increases in inflammation-associated class I/II MHC molecules, CD40,^43^ and immune checkpoint PD-L1^44^ along with increased injury-repair, fibrosis, and stiffness-associated CD44^45–47^ and fibronectin (each of which likely co-localizes with cardiomyocytes rather than being expressed by them).^38^

### The Protein Expression Profile of ‘Granuloma Remote’ CS Parenchymal Tissue Differs from that of Controls:

**Figure 4:**
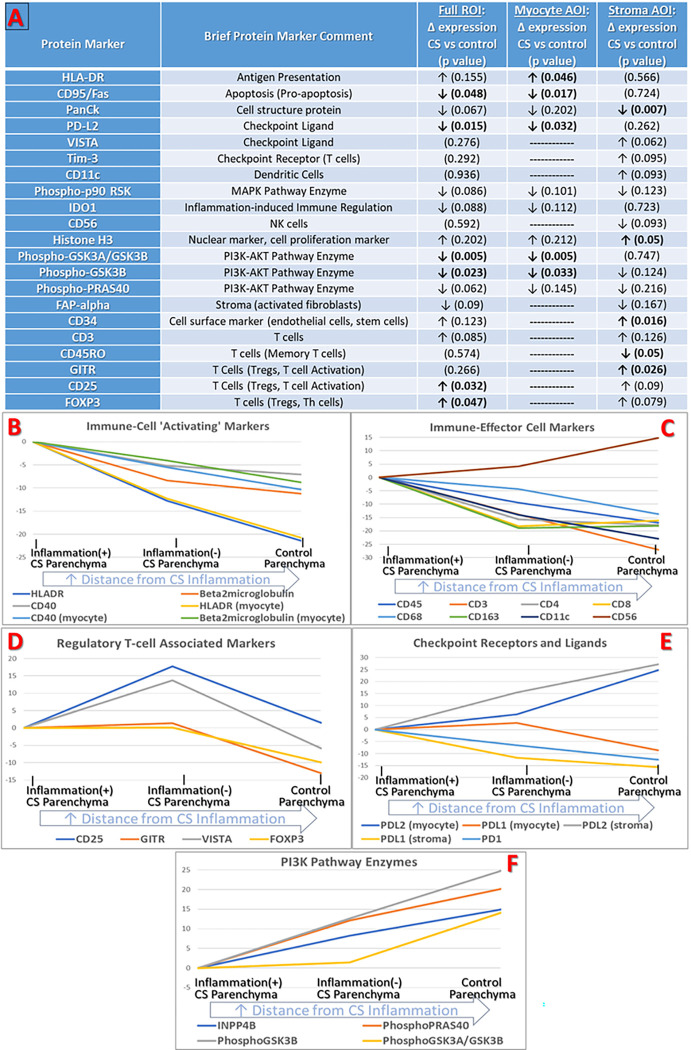
Comparison of cardiac sarcoidosis (CS) cardiac parenchyma and non-CS control tissue parenchyma. **4a:** List of differentially expressed proteins (DEPs) between granuloma-remote, histologic inflammation-free regions-of-interest (ROIs) in cardiac sarcoidosis tissue samples as compared to ROIs from a diverse Control tissue population of failing and non-failing hearts. In addition to ‘full-ROI-level’ differential expression results, digital segmentation of study ROIs enables tissue-compartment-specific analysis of protein expression in discrete ‘areas of illumination’ (AOIs). Via this method, cardiomyocyte-specific and stromal-tissue-specific differential protein expression comparisons between inflammation-free/granuloma-remote parenchymal regions and Control parenchymal regions are also presented. Differentially expressed proteins with p-values <0.1 are shown for full-ROI level data and for myocyte-specific and stroma-specific data. **4b:** Spatial expression results for categories of protein markers, demonstrating change in expression as relative ‘distance’ from CS inflammation increases. Parenchymal tissue regions containing histologic inflammatory infiltrates (‘Inflammation(+) CS parenchyma’) represents the ‘closest’ tissue regions to CS inflammation, ‘inflammation(−) CS parenchyma’ regions are relatively further removed, while ‘Control parenchyma’ regions represent the greatest relative distance from CS inflammation. Different expression trends are noted for different protein marker categories based on distance from inflammation, highlighting both expected findings such as decreased effector immune cell activity with increasing distance, as well as unexpected findings such as variable checkpoint and molecule and regulatory T-cell expression.

A fundamental question at the outset of this research was whether tissue biomarkers of CS exist which can be detected even when no histologic evidence of CS is present. As shown in Fig. 4a and Supplemental Table S.7, we identified numerous significant DEPs between granuloma-remote, inflammation(−), CS tissue and tissue from non-CS controls. Compared to control samples, granuloma-remote CS parenchyma is characterized by significantly increased expression of HLA-DR, Treg markers FOXP3, CD25 and GITR, endothelial/stem-cell marker CD34, and global nuclei/proliferation marker Histone-H3. CS parenchyma manifests decreased expression of CD45RO, PDL2, apoptosis marker CD95/FAS, and *inactivated* (phosphorylated) GSK3β and GSK3α (from which we infer increased *activated* GSK3 enzyme activity with resultant NF-κB-mediated pro-inflammatory cytokine production).^48,49^

To place this finding in the context of the ‘distance-gradient’ results described in the previous section, we performed ordinal logistic regression, treating peri-granuloma regions, granuloma-remote regions, and Control regions as ordinal classes representing different degrees of distance from granulomas. Interestingly, we observed that 65.3% of study panel proteins (n = 49) demonstrated a significant change in expression with increasing distance from CS inflammation (Supplemental Table S.8). Taken together, these results demonstrate both the local impact of granuloma proximity on protein expression as well as the more organ-wide impact of CS on tissue protein expression. Figure 4b provides a further, visual, demonstration of this phenomenon, highlighting the change in expression among several key groups of protein markers when moving from regions of inflammation(+) CS parenchyma to inflammation-free CS parenchyma and finally to control cardiac parenchyma.

A limited, pre-specified sub-analysis of ROIs derived from larger vascular beds was performed to assess whether the vasculature in CS differs from controls. Overall, while ROI numbers were limited for this analysis (n = 29 from CS cases, n = 10 from controls), the results suggest an immunologically active environment in CS vascular beds relative to controls, with increased expression of CD3, CD4, CD68, VISTA, CD45, HLA-DR, and CD11c (Supplemental Table S.9).

### Spatial Protein Expression Biomarkers Enable Accurate Prediction of Occult CS:

**Figure 5:**
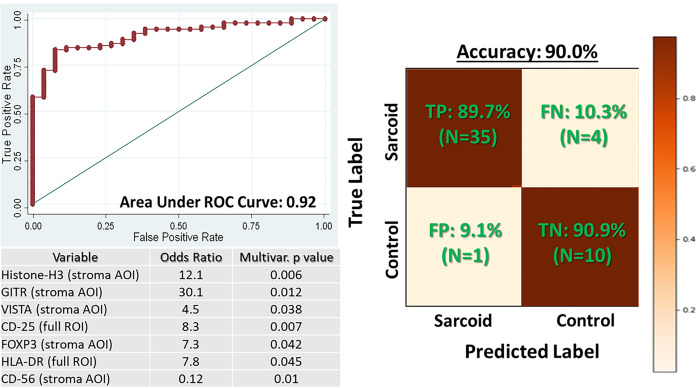
Predictive modeling results for differentiating granuloma-remote, inflammation-free tissue regions from cardiac sarcoidosis (CS) patients vs. controls. Despite being far removed from any conventional histologic evidence of sarcoidosis or active inflammation, the seven protein-expression variables listed in the figure achieve excellent discrimination between CS and control tissue. Receiver operator curve (ROC) represents the leave-one-out cross-validation averaged result during model training on n=107 tissue regions. Confusion matrix results on the right represent performance in a held-out validation set of n=50 tissue regions which were not used during model training.

To assess whether the various DEPs between areas of inflammation(−) CS parenchyma and Controls could have diagnostic value, we developed a binary prediction model to classify parenchyma tissue ROIs as originating from CS vs. Control patients. To maximize clinical utility as a tool capable of improving the diagnostic yield of tissue sampling in CS, the model was specifically developed using *only* data from CS ROIs which were ‘granuloma-remote’ and ‘inflammation(−).’ As shown in Fig. 5, after optimization via leave-one-out cross validation, our final 7-variable logistic regression model was comprised of MHC molecule HLADR, Treg markers FOXP3, CD25, and GITR, immunomodulatory checkpoint molecule VISTA, natural-killer cell marker CD56, and global nuclei/cell proliferation marker Histone-H3. The model achieved excellent performance in the ‘held-out’ validation set, with an accuracy of 90.0%, AUROC of 0.92, sensitivity of 89.7% and specificity of 90.9%. Given that it is also possible to sample peri-granuloma regions during a clinical EMB procedure while still ‘missing’ a granuloma, we also assessed performance of the final model on inflammation(−), peri-granuloma regions. Performance was excellent on these as well, achieving accuracy of 90% (18/20).

## Discussion

In this manuscript, we utilized advanced spatial protein expression profiling, meticulous digital pathology methods, and a unique experimental design to comprehensively study cardiac tissue from CS patients. Our findings provide numerous insights into CS biology, not only within characteristic granulomas, but also within the cardiac parenchymal tissue near granulomas and remote from granulomas. Particularly notable is the discovery of a small set of protein markers which are differentially expressed CS tissue as compared to a diverse group of controls, even when no inflammatory process or granuloma is evident in the surrounding area. This finding has translational value that extends beyond descriptive biology, introducing the possibility of improving the diagnostic yield of biopsies in patients with suspected CS. We believe this report represents an important contribution to mechanistic research in CS, lays the groundwork for a novel precision diagnostic tool, identifies potential therapeutic targets and highlights the translational potential of spatial-omics methods.

### Heterogeneous Immune Profiles of Granulomas in CS:

Molecular heterogeneity of CS granulomas – both within and between samples – is a novel finding in our analysis. Enabled by a cohort with six times as many CS tissues and 23 times as many AOIs as the next-largest spatial profiling study,^13^ our experiments were better equipped to characterize the substantial variability of the CS immune response than any prior investigation. Potential confounders such as varied disease stage and treatment regimens could have contributed to the granuloma protein expression diversity observed during this study. However, neither confounder would explain the marked *intra-sample* heterogeneity we observed, which must necessarily arise from an intrinsic biological mechanism. It is conceivable that CS granulomas develop at different times, and that the relative ‘age’ of a granuloma may dictate the local immune profile. Granulomas may also go through periods of relative activation and quiescence, and thus may present different profiles at different times. There is precedent for granuloma heterogeneity and granuloma ‘aging’ in published tuberculosis research, though no prior CS study has been able to investigate this phenomenon.^50^ In the absence of animal models for CS, prospective cohorts with serial biosampling might be required to provide further mechanistic insights into the causes of granuloma heterogeneity.

### The Immunologically Activated Cardiac Parenchyma in CS:

#### Implications of Disease Stage on Parenchyma Protein Expression:

Our analysis of ‘disease stage’ revealed a large number of DEPs between EMB samples obtained early in the course of symptomatic disease vs. advanced-disease-stage samples obtained at the time of cardiac explant. Though some of these differences could be attributed to treatment effects, the fact that the EMB and explant populations had similar proportions of patients on active treatment at tissue acquisition (14.2% vs. 15.3%) does not support treatment effects as a major confounder. Additionally, though tissue content differences (e.g. large vascular beds, epicardial tissue, fibro-fatty scar, etc.) between endocardial EMB samples and transmural explant samples frequently confound traditional, homogenate-based, ‘omics’ assays, direct visual selection of ROIs as performed in this experiment ensures that unwanted/extraneous tissue areas do not confound our analyses. Thus, we conclude that the progression from active CS to ‘burnt out’ disease is the primary cause for the large number of DEPs observed between EMB and advanced-stage tissue samples. While our results demonstrate that advanced-stage CS is indeed less immunologically ‘active’ than earlier disease, our results also demonstrate that numerous profibrotic which remain quite active in late-stage disease. Cell-therapy clinical studies targeting activated cardiac fibroblasts have received significant attention in recent years,^29^ and our findings suggest that CS may be another potential population for these novel therapeutics.

#### The Distance-Gradient in CS Parenchymal Protein Expression:

Numerous study panel markers were differentially expressed based on a tissue region’s relative distance from CS inflammation. Unsurprisingly, inflammation(+) cardiac parenchyma, which contains overt histologic cellular infiltrates, has increased expression of nearly 2/3rds of the study panel proteins relative to inflammation(−) regions. More interestingly, when comparing inflammation(−) ‘peri-granuloma’ tissue regions to ‘granuloma-remote’ regions, we continued to find significant differential expression of many immune markers and pathways, suggesting that immune activity in CS extends beyond the sites of overt inflammation in a graded, distance-dependent fashion. Whether this observed ‘distance gradient’ is a consequence of recent inflammatory, of paracrine cytokine effects from nearby granulomas, or both, cannot be ascertained without animal models or serial tissue sampling. Nevertheless, it is clear that relative distance from inflammation has meaningful biological effects on the surrounding parenchyma, even when traditional histologic inflammation is absent.

Adding to this narrative is the discovery that even granuloma-remote, inflammation(−) regions in CS hearts manifest in-situ immune profiles that distinguish them from Control tissue. While this finding has obvious diagnostic utility as demonstrated in our predictive modeling efforts, when interpreted in the context of the ‘distance-gradient’ experiments, it also highlights the diffuse nature of parenchymal immune activation in CS. As an example, HLADR manifests significant, graded, differential expression across each of our parenchymal expression experiments. HLADR is increased in inflammation(+) CS tissue relative to inflammation(−) tissue, is increased in peri-granuloma tissue relative to granuloma-remote tissue, and is increased in granuloma-remote tissue relative to Controls. In fact, more than half of our study markers manifest a statistically significant ‘distance gradient’ when peri-granuloma, granuloma-remote, and Control tissue regions are treated as ordinal classes of ‘distance’ from overt CS inflammation. Taken together, these observations lead to the conclusion that while parenchymal immune activation in CS is indeed distance-dependent, it is also sufficiently widespread to enable differentiation of CS tissue from non-CS tissue.

The expression patterns of Treg markers in this experiment represent a particularly interesting manifestation of both widespread and distance-dependent protein expression. Treg and Treg-associated markers FOXP3, CD25, GITR, and VISTA are significantly increased relative to controls in DEP testing and are also incorporated into the CS predictive model. Unlike HLADR which is increased broadly in CS (albeit exhibiting a distance-gradient), these Treg and immune-modulating markers manifest an inverted J-shaped expression pattern in CS tissue, with higher expression in inflammation(−) CS parenchyma than in either inflammation(+) or Control tissue (refer to Fig. 4). Historically, there have been conflicting reports on both the abundance and functional abilities of Tregs in sarcoidosis.^51,52^ It has been speculated that Tregs are functionally deficient in sarcoidosis,^51,52^ and given the serious cardiomyopathy suffered by all cohort patients *in spite* of having elevated Treg-associated protein expression, it is tempting to agree with this theory. However, since our results also show that Treg marker expression is *only* significantly increased in regions *without* overt inflammation, we cannot rule out the possibility that Tregs are preventing the further spread of inflammation into these locations (and thus, are functioning properly). Indeed, given that nearly every T-cell marker *except* FOXP3, CD25, and VISTA, and GITR were significantly increased in inflammation(+) ROIs relative to inflammation(−) ROIs, it seems more likely that a relative insufficiency in Treg number, rather than a deficiency in Treg function, is contributing to inflammatory injury in CS. There is precedence for this theory, with publications on several cardiovascular and autoimmune diseases reporting an association between reduced Treg-to-effector T-cell ratio and pathologic inflammation.^53–55^ This imbalance between the number of Tregs and the number of effector immune cells in inflammation(+) CS parenchyma is only revealed because of the spatial expression methods employed in this experiment. Non-spatially resolved expression methods which would ‘average’ the Treg expression across the tissue, would miss such subtleties in regional expression.

From a translational perspective, the CS predictive model developed in this manuscript proves the existence of broadly expressed tissue biomarkers which can be leveraged to discriminate between CS patients and non-CS patients, even in the absence of nearby granulomas. This finding represents an important step towards a new diagnostic paradigm for CS that is relatively robust despite the patchy nature of granulomatous involvement. While clinical translation of the GeoMx technology used in our experiments is impractical due to cost and technical complexity, there are alternative diagnostic avenues worthy of consideration. Quantitative digital pathology using multi-marker immunostaining and whole-slide image analysis has been piloted in oncologic^56–58^ and cardiovascular research,^14,16^ and represents a more clinically viable methodology. A quantitative immunopathology scoring system based on the small set of CS biomarkers discovered in this report could greatly improve the diagnostic yield of clinical biopsies, enabling earlier intervention and better cohort identification for future therapeutic trials.

As a retrospective cohort study, there are numerous potential confounders which could have impacted study results. Confounding treatment effects may have distorted some of our findings. In addition, though our sample size is larger than any similar study ever conducted, is still modest in absolute terms, limiting statistical power for some subgroup analyses. With respect to our analyses involving ‘granuloma-remote’ tissue regions, it is also conceivable that there are nearby granulomas outside the plane of sectioning which may be impacting our results. However, these occult granulomas hiding in the ‘Z-axis’ would only serve to reduce the statistical significance results, rather than amplify them. Lastly, the nature of the GeoMx assay itself, with a focus on ROIs and digitally segmented AOIs, does not achieve true single-cell-level resolution, limiting interpretation of many finer mechanistic details regarding in-situ CS biology. Follow-up research utilizing prospective sarcoid cohorts, serial tissue sampling (perhaps from other sarcoidosis-affected tissues), complementary single-cell assays, and model-based biological systems would help to address many of these limitations.

## Conclusion

This research represents the largest application of high-plex spatial protein profiling ever performed in human cardiac tissue, and is one of the largest investigations into in-situ CS biology ever conducted. We discovered numerous novel phenomena within CS tissue, providing new insights into granuloma content, disease progression, and the broader parenchymal effects of granulomatous inflammation. Additionally, we leveraged the strengths of ROI-based digital spatial profiling to perform sophisticated biomarker research which has direct translational implications for improving CS diagnostic accuracy. Beyond the specific results of this research, we believe that our approach to cohort design, ROI selection, and digital pathology AOI segmentation comprise an important demonstration of how to best utilize these emerging spatial technologies.

## Figures and Tables

**Table 1:** Cohort details with patient- and tissue-level summary statistics

	Age (yrs)	Race (% Caucasian)	Sex (% Male)	LVEF	Hx of Hypertension	Hx of Diabetes	Hx of Hyperlipidemia	Hx of CKD	Granuloma Present*	Inflammation Present*	ISx at tissue sampling
**Sarcoid (n = 39)**	51.3	53.8%	87.2%	26.1	41.0%	33.3%	28.2%	7.7%	64.1%	53.8%	15.4%
**Biopsy (n = 14)**	50.1	78.6%	85.7%	30.3	42.9%	28.6%	42.9%	0.0%	57.1%	57.1%	14.3%
**Explant (n = 5)**	51.6	54.2%	18.8%	28.6	45.8%	31.3%	31.3%	10.4%	68.0%	52.0%	16.0%
**Control (n = 9)**	52.6	55.6%	55.6%	38.3	66.7%	22.2%	44.4%	22.2%	0.0%	0.0%	22.2%
**Total (n = 48)**	51.6	54.2%	18.8%	28.5	45.8%	31.3%	31.3%	10.4%	64.1%	53.8%	15.4%
**Sarcoid vs Control (p)**	0.739	0.926	**0.029**	**0.025**	0.171	0.527	0.354	0.206	< 0.001	0.003	0.629

## Data Availability

The data that support the findings of this study are presented in the Manuscript and Extended Data sections. Unprocessed raw data is available from the corresponding author upon reasonable request.

## Abbreviations

CS: cardiac sarcoidosis
EMB: endomyocardial biopsy
MRI: magnetic resonance imaging
FDG-PET: Fluorodeoxyglucose positron emission tomography
FFPE: formalin-fixed paraffin embedded
NICM: Non-ischemic cardiomyopathy control
NF: Non-Failing Control
DSP: digital spatial profiling
ROI: region of interest
AOI: area of illumination
Treg: regulatory T cell
MAPK: Mitogen-activated protein kinase associated pathway
PI3K/AKT: Protein kinase B/phosphoinositide-3-kinase/mTOR pathway
DEP: differentially expressed proteins
PCA: principal component analysis
t-SNE: t-distributed stochastic neighbor embedding

## References

[1] Lehtonen J, Uusitalo V, Pöyhönen P, Mäyränpää MI, Kupari M. Cardiac sarcoidosis: phenotypes, diagnosis, treatment, and prognosis. European heart journal. 2023;44(17):1495–1510. 36924191 10.1093/eurheartj/ehad067 PMC10149532

[2] Gilotra NA, Griffin JM, Pavlovic N, Sarcoidosis-Related Cardiomyopathy: Current Knowledge, Challenges, and Future Perspectives State-of-the-Art Review. Journal of Cardiac Failure. 2022;28(1):113–132. 34260889 10.1016/j.cardfail.2021.06.016 PMC8748280

[3] Baughman RP, Valeyre D, Korsten P, ERS clinical practice guidelines on treatment of sarcoidosis. European Respiratory Journal. 2021;58(6):2004079. 34140301 10.1183/13993003.04079-2020

[4] Birnie DH, Nery PB, Ha AC, Beanlands RS. Cardiac sarcoidosis. Journal of the American College of Cardiology. 2016;68(4):411–421. 27443438 10.1016/j.jacc.2016.03.605

[5] Patel MR, Cawley PJ, Heitner JF, Detection of myocardial damage in patients with sarcoidosis. Circulation. 2009;120(20):1969–1977. 19884472 10.1161/CIRCULATIONAHA.109.851352 PMC2778859

[6] Kandolin R, Lehtonen J, Airaksinen J, Cardiac sarcoidosis: epidemiology, characteristics, and outcome over 25 years in a nationwide study. Circulation. 2015;131(7):624–632. 25527698 10.1161/CIRCULATIONAHA.114.011522

[7] Schmidt TJ, Rosenbaum AN, Kolluri N, Natural History of Patients Diagnosed with Cardiac Sarcoidosis at Left Ventricular Assist Device Implantation or Cardiac Transplantation. ASAIO Journal. 2021;67(5). 10.1097/MAT.0000000000001262 33902104

[8] Segura AM, Radovancevic R, Demirozu ZT, Frazier OH, Buja LM. Granulomatous myocarditis in severe heart failure patients undergoing implantation of a left ventricular assist device. Cardiovascular pathology : the official journal of the Society for Cardiovascular Pathology. 2014;23(1):17–20. 23928368 10.1016/j.carpath.2013.06.005

[9] Al-Khatib SM, Stevenson WG, Ackerman MJ, 2017 AHA/ACC/HRS Guideline for Management of Patients With Ventricular Arrhythmias and the Prevention of Sudden Cardiac Death: A Report of the American College of Cardiology/American Heart Association Task Force on Clinical Practice Guidelines and the Heart Rhythm Society. Circulation. 2018;138(13):e272–e391. 29084731 10.1161/CIR.0000000000000549

[10] Ezzat VA, Lee V, Ahsan S, A systematic review of ICD complications in randomised controlled trials versus registries: is our ‘real-world’ data an underestimation? Open heart. 2015;2(1):e000198. 25745566 10.1136/openhrt-2014-000198 PMC4346580

[11] Birnie DH, Sauer WH, Bogun F, HRS expert consensus statement on the diagnosis and management of arrhythmias associated with cardiac sarcoidosis. Heart rhythm. 2014;11(7):1305–1323. 24819193 10.1016/j.hrthm.2014.03.043

[12] Uemura A, Morimoto S, Hiramitsu S, Kato Y, Ito T, Hishida H. Histologic diagnostic rate of cardiac sarcoidosis: evaluation of endomyocardial biopsies. American heart journal. 1999;138(2 Pt 1):299–302. 10426842 10.1016/s0002-8703(99)70115-8

[13] Liu J, Ma P, Lai L, Transcriptional and Immune Landscape of Cardiac Sarcoidosis. Circ Res. 2022;131(8):654–669. 36111531 10.1161/CIRCRESAHA.121.320449 PMC9514756

[14] Peyster EG, Janowczyk A, Swamidoss A, Kethireddy S, Feldman MD, Margulies KB. Computational Analysis of Routine Biopsies Improves Diagnosis and Prediction of Cardiac Allograft Vasculopathy. Circulation. 2022; (in press). 10.1161/CIRCULATIONAHA.121.058459 PMC9133227 35405081

[15] Peyster EG, Arabyarmohammadi S, Janowczyk A, An automated computational image analysis pipeline for histological grading of cardiac allograft rejection. European heart journal. 2021;42(24):2356–2369. 33982079 10.1093/eurheartj/ehab241 PMC8216729

[16] Peyster EG, Wang C, Ishola F, In Situ Immune Profiling of Heart Transplant Biopsies Improves Diagnostic Accuracy and Rejection Risk Stratification. JACC: Basic to Translational Science. 2020;5(4):328–340. 32368693 10.1016/j.jacbts.2020.01.015 PMC7188920

[17] Jeffrey J., Nirschl AJ, Peyster Eliot G., Frank Renee, Margulies Kenneth B., Feldman Michael D., Madabhushi Anant. Deep Learning Tissue Segmentation in Cardiac Histopathology Images. In: Kevin S., Zhou HG, Dinggang Shen, ed. Deep Learning for Medical Image Analysis 2017:pp.179–195.

[18] Chaffin M, Papangeli I, Simonson B, Single-nucleus profiling of human dilated and hypertrophic cardiomyopathy. Nature. 2022;608(7921):174–180. 35732739 10.1038/s41586-022-04817-8 PMC12591363

[19] Tucker NR, Chaffin M, Fleming SJ, Transcriptional and Cellular Diversity of the Human Heart. Circulation. 2020;142(5):466–482. 32403949 10.1161/CIRCULATIONAHA.119.045401 PMC7666104

[20] Chen CY, Caporizzo MA, Bedi K, Suppression of detyrosinated microtubules improves cardiomyocyte function in human heart failure. Nature medicine. 2018;24(8):1225–1233. 10.1038/s41591-018-0046-2 PMC6195768 29892068

[21] Doll S, Dreßen M, Geyer PE, Region and cell-type resolved quantitative proteomic map of the human heart. Nature Communications. 2017;8(1):1469. 10.1038/s41467-017-01747-2 PMC5684139 29133944

[22] van Hijfte L, Geurts M, Vallentgoed WR, Alternative normalization and analysis pipeline to address systematic bias in NanoString GeoMx Digital Spatial Profiling data. iScience. 2023;26(1):105760. 36590163 10.1016/j.isci.2022.105760 PMC9800292

[23] Aibar S, González-Blas CB, Moerman T, SCENIC: single-cell regulatory network inference and clustering. Nature Methods. 2017;14(11):1083–1086. 28991892 10.1038/nmeth.4463 PMC5937676

[24] Liu X. Classification accuracy and cut point selection. Statistics in medicine. 2012;31(23):2676–2686. 22307964 10.1002/sim.4509

[25] Fonseca AC, Melchiades JL, de Campos Soriani Azevedo M, MEK1/2 blockade results in altered inflammatory response and delayed alveolar bone repair. The Journal of Immunology. 2020;204(1_Supplement):73.17–73.17.

[26] Anastasopoulou A, Diamantopoulos PT, Skalioti C, The diagnosis and management of sarcoid-like reactions in patients with melanoma treated with BRAF and MEK inhibitors. A case series and review of the literature. Therapeutic advances in medical oncology. 2021;13:17588359211047349. 34691245 10.1177/17588359211047349 PMC8532252

[27] Kacimi R, Gerdes AM. Alterations in G Protein and MAP Kinase Signaling Pathways During Cardiac Remodeling in Hypertension and Heart Failure. Hypertension. 2003;41(4):968–977. 12642504 10.1161/01.HYP.0000062465.60601.CC

[28] Lemke LE, Bloem LJ, Fouts R, Esterman M, Sandusky G, Vlahos CJ. Decreased p38 MAPK activity in end-stage failing human myocardium: p38 MAPK alpha is the predominant isoform expressed in human heart. Journal of molecular and cellular cardiology. 2001;33(8):1527–1540. 11448140 10.1006/jmcc.2001.1415

[29] Aghajanian H, Kimura T, Rurik JG, Targeting cardiac fibrosis with engineered T cells. Nature. 2019;573(7774):430–433. 31511695 10.1038/s41586-019-1546-z PMC6752964

[30] Serei VD, Fyfe B. The Many Faces of Cardiac Sarcoidosis. American journal of clinical pathology. 2020;153(3):294–302. 31769474 10.1093/ajcp/aqz169

[31] Yoshida T, Hanawa H, Toba K, Expression of immunological molecules by cardiomyocytes and inflammatory and interstitial cells in rat autoimmune myocarditis. Cardiovascular research. 2005;68(2):278–288. 16018993 10.1016/j.cardiores.2005.06.006

[32] Didié M, Galla S, Muppala V, Dressel R, Zimmermann WH. Immunological Properties of Murine Parthenogenetic Stem Cell-Derived Cardiomyocytes and Engineered Heart Muscle. Front Immunol. 2017;8:955. 28855904 10.3389/fimmu.2017.00955 PMC5557729

[33] Herskowitz A, Ahmed-Ansari A, Neumann DA, Induction of major histocompatibility complex antigens within the myocardium of patients with active myocarditis: a nonhistologic marker of myocarditis. J Am Coll Cardiol. 1990;15(3):624–632. 2406319 10.1016/0735-1097(90)90637-5

[34] Ngwenyama N, Kaur K, Bugg D, Antigen presentation by cardiac fibroblasts promotes cardiac dysfunction. Nature cardiovascular research. 2022;1(8):761–774. 10.1038/s44161-022-00116-7 PMC9451034 36092510

[35] Amersfoort J, Eelen G, Carmeliet P. Immunomodulation by endothelial cells — partnering up with the immune system? Nature Reviews Immunology. 2022;22(9):576–588. 10.1038/s41577-022-00694-4 PMC8920067 35288707

[36] Malkova A, Zinchenko Y, Starshinova A, Sarcoidosis: Progression to the chronic stage and pathogenic based treatment (narrative review). Front Med (Lausanne). 2022;9:963435. 36148463 10.3389/fmed.2022.963435 PMC9486475

[37] Patterson KC, Hogarth K, Husain AN, Sperling AI, Niewold TB. The clinical and immunologic features of pulmonary fibrosis in sarcoidosis. Translational research : the journal of laboratory and clinical medicine. 2012;160(5):321–331. 22683422 10.1016/j.trsl.2012.03.005 PMC3910531

[38] Valiente-Alandi I, Potter SJ, Salvador AM, Inhibiting Fibronectin Attenuates Fibrosis and Improves Cardiac Function in a Model of Heart Failure. Circulation. 2018;138(12):1236–1252. 29653926 10.1161/CIRCULATIONAHA.118.034609 PMC6186194

[39] Chi J-Y, Hsiao Y-W, Liang H-Y, Blockade of the pentraxin 3/CD44 interaction attenuates lung injury-induced fibrosis. Clinical and Translational Medicine. 2022;12(11):e1099. 36336784 10.1002/ctm2.1099 PMC9637652

[40] Kuwahara G, Hashimoto T, Tsuneki M, CD44 Promotes Inflammation and Extracellular Matrix Production During Arteriovenous Fistula Maturation. Arteriosclerosis, thrombosis, and vascular biology. 2017;37(6):1147–1156. 28450292 10.1161/ATVBAHA.117.309385 PMC5467640

[41] Sakuishi K, Ngiow SF, Sullivan JM, TIM3(+)FOXP3(+) regulatory T cells are tissue-specific promoters of T-cell dysfunction in cancer. Oncoimmunology. 2013;2(4):e23849. 23734331 10.4161/onci.23849 PMC3654601

[42] Haque ZK, Wang DZ. How cardiomyocytes sense pathophysiological stresses for cardiac remodeling. Cellular and molecular life sciences : CMLS. 2017;74(6):983–1000. 27714411 10.1007/s00018-016-2373-0 PMC6990138

[43] Seko Y, Takahashi N, Azuma M, Yagita H, Okumura K, Yazaki Y. Expression of Costimulatory Molecule CD40 in Murine Heart With Acute Myocarditis and Reduction of Inflammation by Treatment With Anti-CD40L/B7-1 Monoclonal Antibodies. Circulation Research. 1998;83(4):463–469. 9721703 10.1161/01.res.83.4.463

[44] Rodig N, Ryan T, Allen JA, Endothelial expression of PD-L1 and PD-L2 down-regulates CD8+ T cell activation and cytolysis. European journal of immunology. 2003;33(11):3117–3126. 14579280 10.1002/eji.200324270

[45] Weng X, Yue W, Shang L, Inhibition of CD44 attenuates pressure overload-induced cardiac and lung inflammation, fibrosis, and heart failure progression. European heart journal. 2020;41(Supplement_2).

[46] Hellman U, Hellström M, Mörner S, Parallel up-regulation of FGF-2 and hyaluronan during development of cardiac hypertrophy in rat. Cell and Tissue Research. 2008;332(1):49–56. 18196276 10.1007/s00441-007-0562-8

[47] Takeda N, Manabe I. Cellular Interplay between Cardiomyocytes and Nonmyocytes in Cardiac Remodeling. International Journal of Inflammation. 2011;2011:535241. 21941677 10.4061/2011/535241 PMC3175723

[48] Hoffmeister L, Diekmann M, Brand K, Huber R. GSK3: A Kinase Balancing Promotion and Resolution of Inflammation. Cells. 2020;9(4). 10.3390/cells9040820 PMC7226814 32231133

[49] Gong R, Ge Y, Chen S, Glycogen Synthase Kinase 3β: A Novel Marker and Modulator of Inflammatory Injury in Chronic Renal Allograft Disease. American Journal of Transplantation. 2008;8(9):1852–1863. 18786229 10.1111/j.1600-6143.2008.02319.x

[50] Weeratunga P, Moller DR, Ho LP. Immune mechanisms of granuloma formation in sarcoidosis and tuberculosis. The Journal of clinical investigation. 2024;134(1). 10.1172/JCI175264 PMC10760966 38165044

[51] Zhang H, Costabel U, Dai H. The Role of Diverse Immune Cells in Sarcoidosis. Frontiers in Immunology. 2021;12. 10.3389/fimmu.2021.788502 PMC8640342 34868074

[52] Patterson KC, Miller WT, Hancock WW, Akimova T. FOXP3+ regulatory T cells are associated with the severity and prognosis of sarcoidosis. Frontiers in Immunology. 2023;14. 10.3389/fimmu.2023.1301991 PMC10761433 38173720

[53] Wang X, Zhou H, Liu Q, Targeting regulatory T cells for cardiovascular diseases. Front Immunol. 2023;14:1126761. 36911741 10.3389/fimmu.2023.1126761 PMC9995594

[54] Lee GR. The Balance of Th17 versus Treg Cells in Autoimmunity. Int J Mol Sci. 2018;19(3):730. 29510522 10.3390/ijms19030730 PMC5877591

[55] Chung BH, Oh HJ, Piao SG, Clinical significance of the ratio between FOXP3 positive regulatory T cell and interleukin-17 secreting cell in renal allograft biopsies with acute T-cell-mediated rejection. Immunology. 2012;136(3):344–351. 22444300 10.1111/j.1365-2567.2012.03588.x PMC3385034

[56] Lin J-R, Chen Y-A, Campton D, High-plex immunofluorescence imaging and traditional histology of the same tissue section for discovering image-based biomarkers. Nature Cancer. 2023;4(7):1036–1052. 37349501 10.1038/s43018-023-00576-1 PMC10368530

[57] Lee C-W, Ren YJ, Marella M, Wang M, Hartke J, Couto SS. Multiplex immunofluorescence staining and image analysis assay for diffuse large B cell lymphoma. Journal of Immunological Methods. 2020;478:112714. 31783023 10.1016/j.jim.2019.112714

[58] Hoyt CC. Multiplex Immunofluorescence and Multispectral Imaging: Forming the Basis of a Clinical Test Platform for Immuno-Oncology. Frontiers in molecular biosciences. 2021;8:674747. 34150850 10.3389/fmolb.2021.674747 PMC8208831

